# Peer Review of “A Full-Scale Agent-Based Model to Hypothetically Explore the Impact of Lockdown, Social Distancing, and Vaccination During the COVID-19 Pandemic in Lombardy, Italy: Model Development”

**DOI:** 10.2196/32797

**Published:** 2021-09-10

**Keywords:** epidemiology, computational, model, COVID-19, modeling, outbreak, virus, infectious disease, simulation, impact, vaccine, agent-based model

*This is a peer-review report submitted for the paper “A Full-Scale Agent-Based Model to Hypothetically Explore the Impact of Lockdown, Social Distancing, and Vaccination During the COVID-19 Pandemic in Lombardy, Italy: Model Development”.*

## Round 1 Review

### General Comments

This paper [1] is a great effort to apply agent-based modeling to the COVID-19 pandemic. The stated run times for this application are really quite good for such a large number of agents, and I believe that the publication of this paper, when revised, will be very useful to the disease-modeling community.

### Specific Comments

#### Major Comments

1. Though I fully understand how difficult it is to write a scientific paper in a language that is not the primary language of the researchers involved, I am afraid I have to point out that this paper needs some serious revision in its usage of the English language. This is vital to a full understanding of the science applied and the knowledge gained as a result of this work.

2. I would like to see more details about the actual modeling simulation software, algorithms, mathematical functions, etc, used and how it was parallelized/distributed to achieve the efficiency stated in this work. I believe that these application details, rather than its results, are of even greater importance. It is fairly easy to make a model and simulation fit actual outbreak data, so the result is not of much importance when only applied to one set of data. Rather, what is important here is the application methods used to achieve your results as these can be applied to many other epidemiological situations that need to be modeled and simulated.

3. We need much more detail about parts of the model, such as the collision detection algorithm. For example, what was used to determine the result of this portion of the model (eg, what algorithm or mathematical function, etc?)? Please describe the model in detail.

#### Minor Comments

1. I do not believe that a 6-step-per-day model for the agents is too much, contrary to what the authors supposed might be interpreted by the reader. The collision detection and spread caused by an agent's movements throughout the day are likely in great need of many, many steps per day. Further, these “steps” per day could be modified, in future work, to illustrate the effects of movement control or quarantine on the agents of the model.
